# Factors Affecting Level of Physical Activity among Stroke Survivors: A Scoping Review

**DOI:** 10.21315/mjms2024.31.5.8

**Published:** 2024-10-08

**Authors:** Ali Bashir Bakhtiar, Muhammad Hafiz Hanafi, Alia Alghwiri, Haidzir Manaf

**Affiliations:** 1 Centre for Physiotherapy Studies, Faculty of Health Sciences, Universiti Teknologi MARA, Malaysia; 2 University Health Centre, Universiti Malaysia Kelantan, Malaysia; 3 Rehabilitation Medicine Unit, Universiti Sains Malaysia, Malaysia; 4 School of Health and Rehabilitation Sciences, University of Jordan, Jordan

**Keywords:** risk factors, exercise/adverse effects, exercise/classification, exercise/physiology, stroke, sedentary behaviour

## Abstract

Physical activity (PA) is crucial for improving stroke survivors’ health outcomes and quality of life (QoL). Impaired PA levels are common among stroke survivors, a significant portion of whom spend their days in sedentary occupations. Understanding the factors that influence physical inactivity and addressing the barriers to exercise participation can contribute significantly to improving stroke survivors’ health outcomes and prognoses. Therefore, in the current review, we systematically searched five databases (PubMed/Medline, Scopus, SpringerLink, ScienceDirect and Google Scholar) for published studies reporting PA levels among stroke survivors, which yielded 301 potential articles. Based on the identification and screening processes recommended by the Preferred Reporting Items for Systematic Reviews and Meta-Analyses Extension for Scoping Reviews (PRISMA-ScR), 13 articles were finally included in the analysis. The results of these studies, covering 1,318 stroke survivors, revealed physical inactivity among most of the participants and significant heterogeneity among the outcome measures used. The factors affecting PA levels among stroke survivors were mainly categorised as physical, psychological, and sociodemographic, and all were significantly associated with PA levels after strokes.

## Introduction

Strokes are a significant global health concern, ranking as the second leading cause of death and disability worldwide. According to the World Stroke Organization (1), more than 80 million people are currently living with the effects of stroke. Furthermore, the global prevalence of strokes is expected to increase by 25% from 2021 to 2041, primarily due to population ageing and the increasing prevalence of risk factors such as hypertension, diabetes and obesity (2). These statistics highlight the urgent need for effective prevention and management strategies to address the growing burden of strokes.

After having strokes, individuals often face numerous physical, psychological and psychosocial challenges that significantly affect their quality of life (QoL) and overall well-being. Research (3) has shown that, after a stroke, individuals often engage in lower levels of physical activity (PA), leading to various problems, including cognitive impairment. Approximately 38% of stroke survivors experience cognitive decline 1 year after having strokes (4), which can involve memory and orientation impairments, in turn further affecting levels of PA (5, 6). Furthermore, stroke survivors often experience balance disorders, which negatively impact their QoL (7). These impairments, coupled with reduced physical fitness after a stroke, can interfere with daily life activities, exacerbate other stroke-related disabilities, and affect physical functioning, which is crucial for recovery (8, 9). Many stroke survivors experience long-term decreases in physical functioning, including mobility difficulties and an increased risk of falling (10).

Physical inactivity is a common issue among stroke survivors, a significant portion of whom spend their days in sedentary occupations (11). Sedentary behaviour is associated with a higher incidence of cardiovascular disease (CVD) and mortality in the general population (12), and this risk is even greater for stroke survivors, who often face impairments such as muscle weakness, spasticity and cognitive problems (13). These impairments not only contribute to physical inactivity but are also associated with reduced QoL (14). Studies have shown that people’s sedentary behaviour increases after strokes compared to healthy age-matched controls, and that mobility impairments after strokes are associated with more severe strokes (15, 16).

PA plays a crucial role in improving health outcomes and QoL for stroke survivors. Current PA guidelines (17–19) recommend engaging in at least 20 min–60 min of aerobic exercise for 3 days–5 days per week, with an emphasis on minimising sedentary behaviour and incorporating moderate-vigorous physical activity (MVPA) to reap cardiovascular health benefits, regain pre-stroke performance, reduce fatigue and improve health-related quality of life (HRQoL). Research has shown that an active lifestyle improves stroke survivors’ QoL (20). Furthermore, pre-stroke PA is positively associated with post-stroke QoL, underscoring the importance of promoting and maintaining an active lifestyle throughout life (21).

Despite the benefits of PA, not all stroke survivors participate in structured exercise programmes and fewer than 30% meet the minimum PA recommendations (22). Additionally, community-dwelling stroke survivors exhibit lower walking speeds and endurance compared to age-matched controls, and this inactivity is often associated with poor HRQoL, triggering a cycle of debilitation that leads to further limitations, social isolation and impairments in physical fitness (23, 24). Identifying the factors responsible for declining PA among stroke survivors is crucial for improving stroke-related decision-making and prognoses (24). Furthermore, social connections, support and relationships have been shown to influence PA levels in the general older population, which may be relevant for stroke survivors (25).

PA protects against the risk of post-stroke cognitive impairment (PSCI) and vascular dementia among older adults, which cause changes in white matter, highlighting the importance of promoting PA among stroke survivors (26). Post-stroke impairments can lead to increased energy demands and oxygen consumption during daily tasks (27), emphasising the need for customised interventions and support to address physical dysfunction by encouraging PA participation among stroke survivors. Furthermore, stroke survivors exhibit significant differences in their exercise preferences; thus, individual preferences must be considered when promoting PA (28). It is also essential to address barriers to PA and implement community PA programmes for stroke survivors to improve their adherence to PA guidelines and reduce the risk of mortality (20). Despite these challenges and barriers, continuing to stand and move around is vital for stroke survivors and can be achieved with the support and encouragement of caregivers and therapists, who play a crucial role in promoting PA (29). Improving PA among stroke survivors can improve their health outcomes, reduce the risk of mortality and enhance their QoL. Tailored interventions, addressing individual preferences and reducing barriers to participation are crucial for promoting adherence to PA guidelines and maximising the benefits of PA for stroke survivors. Understanding the factors influencing physical inactivity and addressing barriers to exercise participation can contribute significantly to improving stroke survivors’ health outcomes and prognoses. Therefore, we systematically searched for published studies reporting the factors that affect PA levels among stroke survivors. Such a review is warranted to fully understand the nuances of PA engagement in this population.

## Methods

### Protocol

We conducted this scoping review using the methodological framework proposed by Arksey and O’Malley (30).

### Eligibility Criteria

We synthesised the article identification and screening processes using the Preferred Reporting Items for Systematic Reviews and Meta-Analyses Extension for Scoping Reviews (PRISMA-ScR) (31). The inclusion criteria were as follows:

1. original academic articles published in English in peer-reviewed journals
2. cross-sectional/observational/longitudinal/cohort/secondary studies investigating stroke survivors (acute/chronic stroke patients) and the factors affecting their PA levels after strokes.

We included all studies except systematic and narrative reviews, dissertations and technical papers, and we also included reports on PA levels and the factors influencing them. We excluded studies that did not report on PA and articles that only reported the protocols for interventions. We were interested in the factors influencing PA that could be objectively measured (e.g. with accelerometers or pedometers) or subjectively measured or graded by assessors.

### Search Strategy

Between 20 May 2023 and 24 May 2023, we conducted a comprehensive search of five electronic databases (PubMed/Medline, Scopus, SpringerLink, ScienceDirect and Google Scholar) for research articles that met the inclusion criteria and were published in English. We used combinations of keywords and Boolean logic commands (‘AND’, ‘OR’ and ‘NOT’) related to the theory (factors), population of interest (stroke survivors) and context (PA). The results were sorted by relevance, as shown in Table 1.

### Data Extraction and Analysis

Two reviewers independently screened all references and retrieved full-text articles to identify eligible articles. Discrepancies between the reviewers were resolved via consensus. Subsequently, the selected articles underwent data extraction by the same two reviewers, who meticulously input the information into a structured Excel sheet for tabulation. The extracted data encompassed the name(s) of the author(s), year of publication, country, subject, methodology (period, procedure and contact time), factor outcome measures, PA level outcome measures and study findings.

To ensure the accuracy and reliability of the data, two other reviewers meticulously scrutinised the Excel sheet to identify and rectify any errors in the data extraction process. The data were then synthesised and summarised based on the methodological approaches of the included studies, the characteristics of the subjects under investigation, the outcome measures of the investigated factors and their influence on PA levels. This rigorous process provided a comprehensive and reliable analysis of the gathered data, ensuring that the findings were robust and trustworthy.

## Results

**Figure 1:**
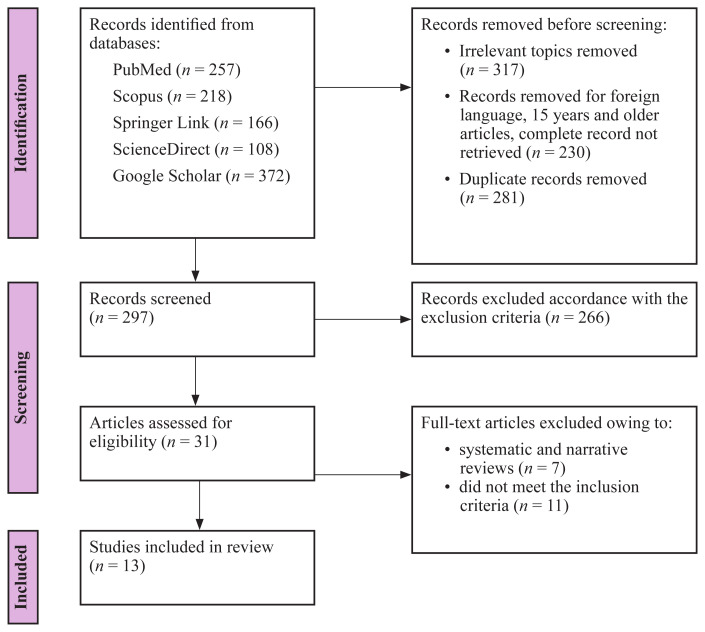
PRISMA-ScR flow diagram

A PRISMA-ScR flow diagram of the article screening and selection stages is shown in Figure 1. A total of 297 articles were identified from databases and other sources, including Google, of which 266 were excluded after screening the titles and abstracts. Further full-text screening led to the subsequent removal of 18 articles, leaving 13 articles eligible for data extraction.

### Study Description

After the reviewers comprehensively analysed the data, we categorised the identified factors as physical psychological and sociodemographic factors, and tabulated them as shown in Tables 2, 3 and 4, respectively. Overall, the studies assessed 1,318 stroke survivors. The sample sizes ranged from 40 to 215. Of the 13 reviewed studies, only 3 studies employed small sample sizes that limited the generalisability of their results (32–34). The duration of the studies ranged from 7 days to 3 years. Six studies were conducted in Sweden (24, 30, 35–38) and the others in Norway, Singapore, Australia, South Africa, the United States, West Africa and Spain (33, 34, 39–43).

The stroke populations in the reviewed studies varied. Two studies were conducted among chronic stroke survivors (41, 43), three studies considered first-stroke survivors and three studies investigated community-dwelling stroke survivors (34, 37–40, 42). Three other studies were conducted among inpatient stroke patients who received rehabilitation treatment (32, 33, 35). The remaining two studies considered stroke patients who received treatment in university hospitals (24, 36).

Almost all the studies applied one-time assessments for data collection (24, 35, 37, 38, 41–43), although a study applied two assessments (after 3 and 12 months and 7 days prior to discharge and 3 months following discharge) (33, 40). This pattern was also followed by one study that involved an initial assessment followed by a second assessment a year later (36). Another study relied on a different approach—daily follow-up during 4 weeks of inpatient rehabilitation and further follow-up via home visits 4 weeks after completion of outpatient rehabilitation (36). A further study applied three assessments of 3 months, 6 months and 12 months post-stroke (32) and another assessed participants for 5 days during a week from Monday to Friday (34).

### Factors Associated with PA Levels

None of the reviewed studies revealed significant associations between the investigated factors and PA levels. However, the associations between the investigated factors differed in each study. Zalewski and Dvorak (34) discovered that physical ability was significantly associated with PA levels, while Påhlman et al. (36) found that cognitive performance and executive performance were associated with PA levels. However, Vahlberg et al. (38), in a cross-sectional cohort study, found strong associations between mobility, fall-related efficacy, and PA. Persson et al. (32) investigated the association between postural control and PA levels and discovered a significant association. Joseph et al. (42), in a cross-sectional study, found that age, stroke severity, and failure to receive outpatient rehabilitation were associated with lower PS levels.

Fini et al. (39) investigated PA levels following strokes and indirectly found that age, gait, speed, and cognitive performance were associated with PA levels. However, physical functioning (PF) upon discharge, level of motivation, and levels of depression, stress and anxiety were found to be significantly associated with lower PA levels (33). Persson and Hansson (37), in a 6-month study of factors associated with PA, discovered that stroke severity, physical inactivity before stroke, number of drugs used during active stroke and postural control were highly associated with PA levels. Hamre et al. (40) conducted a longitudinal observational cohort study and discovered that pre-stroke sick leave, neuropsychiatric symptoms, and post-stroke balance performance were significantly associated with lower PA levels. However, Viktorisson et al. (24) found that only cognitive performance was significantly associated with PA levels.

Botö et al. (35) considered admitted stroke patients and discovered that stroke severity, physical inactivity before stroke, and fear of falling were significantly associated with PA levels. Sanchez-Sanchez et al. (43) conducted a cross-sectional study and showed that walking speed and physical barriers influenced PA levels; overall step counts were highly associated with PA levels among chronic stroke survivors.

### Outcome Measures of the Investigated Factors

#### Stroke Severity

Four studies (24, 36, 40, 42) used the National Institute of Health Stroke Scale (NIHSS) to quickly assess stroke severity.

#### Neuropsychiatric Symptoms

Each study employed specific outcome measures according to the investigated factors. Four studies (33, 39, 40, 42) screened for neuropsychiatric symptoms and most used the Hospital Anxiety and Depression Scale (HADS). However, two studies used the Geriatric Depression Scale (GDS) and the 9-Question Patients Health Questionnaire (PHQ-9), respectively. owing to the populations they considered (38, 43).

#### Cognitive Functioning

For cognitive screening, the Montreal Cognitive Assessment (MoCA) was widely used in most of the reviewed studies, with only one study using the Mini-Mental State Examination (MMSE) (24, 37, 39). Hamre et al. (40), Joseph et al. (42) and Vahlberg et al. (38) employed the Trail Making Test (TMT), MMSE and Short Portable Mental Status Questionnaire (SPMSQ) for cognitive screening, respectively.

#### Postural Control and Balance

We observed a heterogeneity in the utilisation of outcome measures for postural control and balance assessment. The Swedish version of the Postural Assessment Scale for Stroke (SwePASS) was favoured by three studies (32, 35, 37). Botö et al. (35) seemed to use two outcome measures for balance assessment—the SwePASS and the Berg Balance Scale (BBS)—whereas two studies (40, 42) employed the Balance Evaluation System Test (BESTest).

#### Physical Ability (Gait Speed and Walking Capacity)

Physical ability and functioning were assessed using walking parameters. Most studies used the 10 Metre Walk Test (10MWT) and the Six Minute Walking Test (6MWT). Several used the 10MWT and 6MWT to assess gait speed and physical functioning (33, 34, 39, 43). However, Honado et al. (41) only used step counts to measure the overall steps and physical abilities of the participants.

#### Fatigue

Three studies (39, 40, 43) used the Fatigue Severity Scale (FSS) to measure levels of fatigue among their subjects.

#### Disability and Functional Outcomes

Three studies employed the modified Rankin Scale (mRS) to assess levels of dependence after stroke (24, 36, 43).

#### Fall

Four studies (33, 38, 42, 43) used the Falls Efficacy Scale (FES) to measure subjects’ ability to perform ADL without falling.

#### Ambulation Category

Only two studies (24, 39) used the Functional Ambulation Classification (FAC) to measure participants’ walking ability.

#### Quality of Life

Only Vahlberg et al. (38) investigated the association of QoL with PA levels using the European QoL-5 Dimension-3 Level (EQ-5D-3L).

#### Barriers to Physical Activity

Sanchez et al. (43) and Zalewski and Dvorak (34) assessed barriers to PA using the Barriers to Physical Activity after Stroke Scale (BAPAS) and the Barriers to Being Active Quiz (BBAQ), respectively.

### Other Outcome Measures

One study (32) used the Modified Motor Assessment Scale, Uppsala Akademiska Hospital-95 (M-MAS UAS-95) to simultaneously measure mobility, postural control, walking and upper limb (UL) functioning among stroke survivors. Joseph et al. (42) used the Barthel Index (BI) and the Timed Up and Go Test (TUGT) to assess participants’ ADL and mobility, respectively. Thilarajah et al. (33) assessed the participants’ muscle strength using isometric muscle testing and behaviour regulation during exercise using the Behavioural Regulation in Exercise Questionnaire (BREQ). Only Hamre et al. (40) measured levels of apathy using the Apathy Evaluation Scale (AES), and Sanchez-Sanchez et al. (43) assessed physical functioning using the Stroke Impact Scale (SIS).

### Outcome Measures for Physical Activity Levels

Four reviewed studies (24, 35–37) used the Saltin-Grimby Physical Activity Level Scale (SGPALS) to measure PA levels, but two (32, 38) preferred the Physical Activity Scale for the Elderly (PASE). Many of the reviewed studies (34, 39, 42, 43) used accelerometers to measure step counts and define the PA levels of their subjects. Only one study (41) used step counts to calculate PA levels based on energy expenditure. Thilarajah et al. (33) used a combination of the Activity Card Sort (ACS) and the International Physical Activity Questionnaire (IPAQ) to determine the PA levels of their subjects, whereas Hamre et al. (40) adapted a self-report questionnaire from a previous study—the Nord-Trondelag Health Study (HUNT)—to assess PA.

### Levels of Physical Activity

Most of the reviewed studies (24, 34–37, 42, 43) that examined the PA levels of their subjects reported that they were largely physically inactive. Only four studies (32, 33, 39, 40) revealed that most of their participants were physically active, and two (38, 41) did not report on PA.

## Discussion

In the current scoping review, we examined 13 articles related to the determinants of PA among stroke survivors. It was clear that three categories of factors—physical, psychological and sociodemographic—primarily contribute to physical inactivity among stroke survivors, with physical factors being the most influential. Eight out of the 13 articles reported associations between physical factors and post-stroke PA levels. However, regarding the underlying causes of the factors involved, researchers should go beyond focusing on the established reasons provided by the reviewed articles to gain a holistic understanding of the investigated issue.

The most important aspect of the data was the association between physical factors and post-stroke PA levels. PA is crucial for enabling stroke survivors to improve their health outcomes and QoL, including improved cardiovascular health, regained pre-stroke performance, reduced fatigue and improved HRQoL (44). Research has shown that PA can have numerous benefits for stroke survivors. For example, exercising for at least 20 min–60 min, 3–5 days per week, is associated with these four cardiovascular health benefits (17–19). One study (20) found that an active lifestyle can significantly improve the QoL of stroke survivors. Furthermore, PA has been positively associated with post-stroke QoL, highlighting the importance of promoting and maintaining an active lifestyle throughout life (21). Despite the numerous benefits of PA for stroke survivors, it is important to recognise that not all individuals are able or willing to participate in structured exercise programmes, possibly due to various barriers and challenges, such as physical limitations, fear of injury, lack of motivation or limited access to suitable exercise facilities. A study has shown that fewer than 30% of stroke survivors meet minimum PA recommendations (22).

Declining walking speed and endurance among community-dwelling stroke survivors compared to age-matched controls has significant consequences for overall health and well-being (23). This decline in PA is often associated with poor HRQoL and can trigger a cycle of debilitation, leading to social isolation and further physical fitness limitations and impairments (23, 24). For stroke survivors, the ability to walk independently and maintain functional mobility is crucial for daily activities and community participation. Identifying the factors responsible for decreased PA among stroke survivors is crucial for improving stroke-related decision-making and prognoses (24). In addition, social connections, support and relationships influence PA levels in the general older population, which could also be relevant for stroke survivors (25).

Psychological factors, such as neuropsychiatric symptoms and cognitive impairment, pose considerable problems for stroke survivors. Interestingly, 3 of the 13 reviewed articles (24, 36, 39) reported that cognitive impairments were significantly associated with lower post-stroke PA levels. The researchers interpreted this finding as being due to cognitive impairment and declining cognitive functioning during the first 6 months after stroke hindering PA participation and leading to low PA levels. Recent evidence (26) has shown that cognitive impairments are indeed likely to cause restrictions and dependence on ADL among stroke survivors, potentially limiting activities and jeopardising the recovery process. Therefore, preservation of pre-stroke cognition is essential for enabling stroke survivors to achieve higher PA levels following strokes (36). Meanwhile, other studies (33, 40) have shown that levels of depression, anxiety and stress can predict post-stroke PA levels. The authors (33, 40) explained that this is because post-stroke changes, such as fatigue and depression resulting from post-stroke impairments and existing co-morbidities, are likely to cause physical inactivity and negatively affect physical functioning. These findings corroborate a previous review by Thilarajah et al. (33), which showed that cardiorespiratory fitness, walking endurance, comfortable gait, speed, balance, fall effectiveness, depression and HRQOL were significantly associated with PA levels. In addition, individuals who have experienced stroke may become apathetic, losing interest in activities and exhibiting reduced motivation (45). Therefore, there is an association between post-stroke depression and motivation that later contributes to reduced attention and memory performance and leads to physical disturbance after a stroke (46). It is essential to maintain and not reduce PA to alleviate neuropsychiatric symptoms after a stroke (47).

Age, stroke severity, pre-stroke PA and drugs administered during the acute phase are characteristic sociodemographic factors associated with post-stroke PA levels (35, 37, 39, 42). Joseph et al. (42) and Fini et al. (39) found that age was associated with PA levels, but they claimed that differences between physical capabilities and the definition of MVPA itself influenced the results. In addition, PA gradually declines with age as people lose muscle mass and strength, with PA often decreasing by 40%–80%, increasing the probability of developing metabolic disorders or chronic diseases (48). A new study by Roaldsen et al. (49) revealed that stroke survivors who live in the community are less active than healthy peers of their age, and many of them do not follow PA recommendations, thus increasing their risk of having another stroke or CVD. Studies have found stroke severity to be associated with lower PA levels (35, 37, 42). Joseph et al. (42) claimed that this is due to the lack of systematic post-stroke rehabilitation in developing countries, such as South Africa, hindering the promotion of PA after stroke. Although Persson and Hansson (37) speculated that immobilised individuals would be at high risk of becoming inactive even if they tried to remain physically active, they selected a study population with extremely low stroke severity. Botö et al. (35) explained that a severe stroke can cause neurological symptoms, such as paresis, potentially leading to reduced mobility and a subsequent risk of physical inactivity. Recent evidence (50) supports these findings, revealing that PA strongly predicts milder stroke severity. Persson and Hansson (37) and Botö et al. (35) found that pre-stroke physical inactivity was associated with low PA levels after strokes. However, they explained that there was no association among stroke survivors between low physical inactivity, post-stroke PA levels, and greater stroke severity, mainly because higher pre-stroke PA is associated with longer-term light PA, MVPA, and less time spent sitting (44). Furthermore, Persson and Hansson (37) discovered that the number of drugs used during the acute stroke phase was associated with lower PA levels. They suggested that drugs administered to patients during the acute phase served as a proxy for comorbidity, which, in turn, altered the participants’ PA performance going forward (37). Consistent with this argument, Clarke and Witham (51) stated that medications may limit patients’ PA by worsening existing symptoms, which are often side effects of the treatment of another disease.

Several beneficial findings of the current review are relevant to clinical practice and future research. Based on the findings, professionals and clinicians clearly play a vital role in post-stroke care, especially in the management of post-stroke PA. The prioritisation of recommended levels of PA among stroke survivors could be intensified and PA should be emphasised for patients with stroke risk factors to break up sitting time and reduce the risk of high stroke severity (52). Furthermore, objective measures could be used for post-treatment assessments of stroke survivors to eliminate inaccurate treatments, and clinicians should scrutinise patients’ medication histories to ensure that possible side effects that could disrupt their recovery processes are identified. Another suggestion for professionals is to ensure a systematic approach to rehabilitation among stroke survivors, especially chronic and community-dwelling stroke survivors, to ensure adequate follow-up and maintenance of adequate PA levels.

Future researchers should not only focus on a thorough exploration of the factors considered in this review, but also investigate new factors. Additionally, future researchers should consider several other relevant domains to achieve more holistic findings. Future researchers could also consider more objective outcome measures to ensure more valid and accurate outcomes.

Generally, the present study provides a clear understanding of the influence of physical, psychological and sociodemographic factors on PA levels following strokes for researchers and professionals. In addition, it can update the pool of knowledge underpinning stroke studies. However, the present study has several limitations. We observed a wide range of study durations, methodologies and outcome measures used to measure PA levels, and some of the reviewed articles mixed two or three factors, which led to widespread heterogeneity and made meta-analysis impossible. Second, we did not formally evaluate the methodological quality and risk of bias of the included studies, as the general purpose of the scoping review was to provide a more comprehensive overview of the available evidence. Finally, despite our extensive search of the literature, the number of reviewed studies was small, limiting the strength of the conclusions.

## Conclusion

Few studies have investigated the factors affecting PA levels among stroke survivors. After reviewing 13 studies, several factors were significantly associated with and influenced stroke survivors’ PA levels to various extents, such as age, severity of stroke, sedentary behaviour, neuropsychiatric symptoms, post-stroke cognitive level, walking speed, ability to walk, balance, postural control, level of motivation and mobility. These findings contribute to the knowledge pool in this study area, serve as a reference for professionals by providing strong evidence and offer additional information to stroke caregivers in terms of key success factors associated with improvements in PA levels during stroke rehabilitation.

## Figures and Tables

**Table 1:** Search strategies used in the current review

Search strategy items details	Details
Used keywords	Combination of two from the following: - Factors* OR related constructs (e.g. determinants or risk factors) - Physical activity* OR exercise* OR Sport - Stroke* OR Stroke survivors* OR Stroke patients*
Searched databases	PubMed/Medline, Scopus, Springer Link, ScienceDirect and Google Scholar
Time filter	*none*
Language filter	English only
Document type filter	Articles in peer-reviewed journals
Inclusion criteria	Original article complying to the established PICO
Exclusion criteria	Reviews, Dissertation, Technical papers

Note: PICO = Population, Intervention, Comparison and Output

**Table 2:** Physical factors association with post-stroke physical activity

Author/Year	Country	Subject (*n*)	Type of study	Methodology			Outcome measure on investigated factors	Findings (outcome measure of PA)

				Period	Procedure	Contact hour		
Zalewski and Dvorak/2011	United State	Community-dwelling older adults stroke survivors and their care partners (*n* = 40)	Cross-sectional study	7 days	PA were measured continuously for 3 days using accelerometer, physical ability testing was performed on 200-meter indoor track and questionnaire were administered via in person or telephone call	Monday–Thursday	6MWT, 10MWT, BBAQ	Participants with stroke were classified as sedentary, averaging 2,990 (± 2,488) steps per day. (7-day PAR) For stroke survivors, 6MWT and 10MWT: ↑↓ For care partners, 6MWT and 10MWT: ↔
Vahlberg et al./2013	Sweden	Community-living individuals with a previous stroke (*n*= 187)	Cross-sectional cohort study	1–3 years	Self-administered questionnaires, F2F interview and performance-based measurement	One-time F2F interview and performance-based measurement	FES-S, EQ-5D, GDS, SPMSQ	Self-reported PA was associated with walking speed in men. The regression model explained 29% of the variance in the PASE outcome. In women, self-reported PA was associated with balance, as measured by the BBS. The regression model explained 23% of the variance in the PASE outcome (PASE) Mobility and fall-related self-efficacy: ↑↓
Persson et al./2016	Sweden	People admitted to the stroke unit consecutively (*n*= 96)	Observational study	12 months	Participants were assessed for self-reported physical activity and postural control using PASE and SwePASS, respectively during 3, 6 to 12 months post-stroke.	Three consecutive appointments on 3, 6 and 12 months after stroke	SwePASS, M-MAS UAS-95	Self-reported PA was associated with walking speed in men. The regression model explained 29% of the variance in the PASE outcome. In women, self-reported PA was associated with balance, as measured by the BBS. The regression model explained 23% of the variance in the PASE outcome (PASE) Mobility and fall-related self-efficacy: ↑↓ The raw median PASE scores at 3, 6, and 12 months after stroke were 59.5, 77.5, and 63.5, respectively. Between 3 and 6 months after stroke, PASE scores increased by 32%, with no significant change between 3 and 12 months and between 6 and 12 months after stroke. (PASE) Postural control: ↑↓
Fini et al./2021	Australia	First ever stroke patients admitted to Australia metropolitan rehabilitation hospital (*n* = 79)	Prospective longitudinal observational study	1 month	All participants were asked to wear an accelerometer for 7 days (5 days including 1 day on weekend) and continue with their daily activities.	4 weeks during inpatient rehabilitation or 4 weeks upon completion of outpatient rehabilitation or home-visit assessment	Accelerometer, 10MWT, 6MWT, FAC, MoCA, FSS, HADS	Twenty participants (25%) did not meet either the daily step or MVPA targets recommended. (Accelerometer) Participants age: ↑↓ Gait speed: ↑↓ Cognitive level: ↑↓
Sanchez et al./2021	Spain	Chronic stroke survivors (*n* = 57)	Cross-sectional study	7 days	Comprises of F2F interviews, physical assessments, and questionnaires administration	One-time appointment right after the recruitment	MoCA, mRS, PHQ-9, SIS-16, 10MWT, Short FES-I, BAPAS, FSS, Accelerometer	Fifty-seven participants (58.2 ± 11.1 years, 37 men) met the accelerometer wear–time criteria (three days, ≥eight h/day) (Accelerometer) Walking speed and physical barriers: ↑↓
Hamre et al./2021	Norway	Hospitalized patients in acute stroke units (*n* = 101)	Observational longitudinal cohort study	12 months	One-time session of interviews, patients record screening and physical examination	Appointment on early 3 months and on the 12^th^ month	BESTest, TMT-B, HADS, AES-S, FSS	Almost 80% of the population were physically active (HUNT Self-Reported Questionnaire) Post-stroke balance: ↑↓ Neuropsychiatric symptoms: ↑↓
Boto et al./2021	Sweden	Patients admitted to stroke unit (*n* = 190)	Prospective longitudinal cohort study	12 months	Assessment was performed using questionnaires	One-time assessment after 12 months registration at the stroke unit	BBS, SwePASS	Before stroke onset, 50% of participants reported being physically inactive. After 1 year, the corresponding proportion was 37%. (SGPALS) Physical inactivity before stroke, stroke severity and fear of falling: ↑↓
Honado et al./2023	West Africa	Stroke survivors and able-bodied adults (*n* = 120)	Secondary analysis	7 days	Involving 7 days step counting and questionnaire administration	One-time assessment	Step counts	(IPAQ-AF) Overall step counts: ↑↓

Notes: BESTest = Balance Evaluation System Test; TMT-B = Trail Making Test Part B; HADS = Hospital Anxiety and Depression Scale; AES-S = Apathy Evaluation Scale-Self Reported; HUNT = Health Study in Nord-Trandelag; FAC = Functional Ambulation Category; FSS = Fatigue Severity Scale; 10MWT = 10 Meter Walking Test; FES = Falls Efficacy Scale; PA = Physical Activity; 6MWT = 6 Minutes Walking Test; MoCA = Montreal Cognitive Assessment; SwePASS = Swedish Postural Assessment Scale for Stroke; MAS UAS-95 = Modified Motor Assessment Scale, Upsala Akademiska Hospital-95; MVPA = Moderate to Vigorous Physical Activity; IPAQ-AF = International Physical Activity Questionnaire-French version; SGPALS = Saltin-Grimby Physical Activity Level Scale; PASE = Physical Activity Scale for Elderly; EQ-5D-3L = European Quality of Life-5 Dimension-3 Level Questionnaire; FES-I = Falls Efficacy Scale (International); BBAQ = Barriers to Being Active Quiz; mRS = modified Rankin Scale; GDS = Geriatric Depression Scale; SPMSQ = Short Portable Mental Status Questionnaire; F2F = Face to Face; SwePASS = Swedish Postural Assessment Scale for Stroke; BBS = Berg Balance Scale; PHQ-9 = Patient Health Questionnaire-9; SIS-16 = Stroke Impact Scale-16; BAPAS = Barriers to Physical Activity After Stroke Scale

**Table 3:** Psychological factors association with post-stroke physical activity

Author/Year	Country	Subject (*n*)	Type of study	Methodology			Outcome measure on investigated factors	Findings (outcome measure of PA)

				Period	Procedure	Contact hour		
Pahlman et al./2012	Sweden	Patient admitted to university hospital stroke unit (*n* = 74)	Prospective study	1 year	Involving medical and neurological examination per individual	One initial appointment (1st appointment) followed by 2nd appointment 1 year after	NIHSS, mRS	Participants with stroke were classified as sedentary, averaging 2,990 (± 2,488) steps per day (Accelerometer) Cognitive performance and executive function: ↑↓
Fini et al./2021	Australia	First ever stroke patients admitted to Australia metropolitan rehabilitation hospital (*n* = 79)	Prospective longitudinal observational study	1 month	All participants were asked to wear an accelerometer for 7 days (5 days including 1 day on weekend) and continue with their daily activities	Four weeks during inpatient rehabilitation or Four weeks upon completion of outpatient rehabilitation or home-visit assessment	Accelerometer, 10MWT, 6MWT, FAC, MoCA, FSS, HADS	Twenty participants (25%) did not meet either the daily step or MVPA targets recommended (daily steps) Participants age: ↑↓ Gait speed: ↑↓ Cognitive level: ↑↓
Thilarajah et al./2020	Singapore	Patients from inpatient rehabilitation unit (*n* = 64)	Prospective cohort study	3 months	Two assessments comprised of physical examination and questionnaires	Seven days prior of discharge from inpatient rehabilitation unit (1st assessment) and 3 months following discharge (2nd appointment)	10MWT, Isometric muscle testing, HADS, FES, BREQ-2, ACS	At 3 months after discharge from rehabilitation, survivors of stroke took a median (IQR) of 4,870 (1,904–8,885) steps/day, with a wide range of individual daily counts from 58 to 16,643 steps/day (ACS and IPAQ) Level of motivation: ↑↓ Level of depression, anxiety and stress: ↑↓
Viktorisson et al./2021	Sweden	Stroke patients receiving treatment at university hospital (*n* = 49)	Prospective longitudinal exploratory study Observational longitudinal cohort study	6 months	Participants were recruited at 2 days–15 days after stroke, and screened for cognitive impairments, information on pre-stroke PA was retrospectively collected during admittance and poststroke PA was evaluated after 6 months	Six months after post-stroke	MoCA, FAC, NIHSS, mRS	The level of physical activity changed in more than half of all participants after stroke Participants who were physically active 6 months after stroke presented with significantly less cognitive impairments. (SGPALS) Cognitive impairment: ↑↓
Hamre et al./2021	Norway	Hospitalised patients in acute stroke units (*n* = 101)	Prospective longitudinal exploratory study Observational longitudinal cohort study	12 months	One-time session of interviews, patients record screening and physical examination	Appointment on early 3 months and on the 12th month	BESTest, TMT-B, HADS, AES-S, FSS	Almost 80% of the population were physically active (HUNT Self-Reported Questionnaire) Post-stroke balance: ↑↓ Neuropsychiatric symptoms: ↑↓

Notes: BESTest = Balance Evaluation System Test; TMT-B = Trail Making Test Part B; AES-S = Apathy Evaluation Scale-Self Reported; HADS = Hospital Anxiety and Depression Scale; 10MWT = 10 Meter Walking Test; FES = Falls Efficacy Scale; FSS = Fatigue Severity Scale; HUNT = Health Study in Nord-Trandelag; BREQ-2 = Behavioural Regulation in Exercise Questionnaire-2; ACS = Activity Card Sort; IPAQ = International Physical Activity Questionnaire; PA = Physical Activity; FAC = Functional Ambulation Category; MoCA = Montreal Cognitive Assessment; NIHSS = National Institute of Health Stroke Scale; mRS = modified Rankin Scale; MVPA = Moderate to Vigorous Physical Activity, SGPALS = Saltin-Grimby Physical Activity Level Scale

**Table 4:** Sociodemographic factors association with post-stroke physical activity

Author/Year	Country	Subject (*n*)	Type of study	Methodology			Outcome measure on investigated factors	Findings (outcome measure of PA)

				Period	Procedure	Contact hour		
Joseph et al./2018	South Africa	Community-dwelling stroke survivors (*n* = 45)	Cross-sectional study	3 days–5 days	Comprises of structural interviews of contextual factors, clinical tests of body functions and activity and objective measurement of PA	One-time interview, clinical test and measurement	Barthel Index, Mini-BESTest, TUGT, 6MWT, FES-I, HADS, MMSE, NIHSS	The median number of steps per day was 2,393 and of the average 703 min of wear time, 80% were spent in sedentary, 15% in light and 5% in moderate-to-vigorous intensity physical activity (Accelerometer) Age, stroke severity and failing to receive outpatient rehabilitation: ↑↓
Fini et al./2021	Australia	First ever stroke patients admitted to Australia metropolitan rehabilitation hospital (*n* = 79)	Prospective longitudinal observational study	1 month	All participants were asked to wear an accelerometer for 7 days (5 days including 1 day on weekend) and continue with their daily activities	Four weeks during inpatient rehabilitation or 4 weeks upon completion of outpatient rehabilitation or home-visit assessment	Accelerometer, 10MWT, 6MWT, FAC, MoCA, FSS, HADS	Twenty participants (25%) did not meet either the daily step or MVPA targets recommended (daily steps) Participants age: ↑↓ Gait speed: ↑↓ Cognitive level: ↑↓
Persson and Hansson/2020	Sweden	First ever stroke patients (*n* = 215)	Prospective longitudinal cohort study	6 months	Baseline assessment were performed using questionnaires	Four days post-admission to stroke unit	SwePASS, MoCA	The physical BAPAS score explained 28.7% of the variance of the prolonged sedentary time (β = 0.547; *P* < 0.001). Additionally, the walking speed (β = 0.452) together with physical BAPAS (β = −0.319) explained 37.9% of the moderate-to-vigorous PA time (*P* < 0.001) (Accelerometer) Stroke severity, physical inactivity before stroke, moderate postural control, poor postural control and number of drugs in acute stroke: ↑↓
Boto et al./2021	Sweden	Patients admitted to stroke unit (*n* = 190)	Prospective longitudinal cohort study	12 months	Assessment were performed using questionnaires	One-time assessment after 12 months registration at the stroke unit	BBS, SwePASS	Before stroke onset, 50% of participants reported being physically inactive. After 1 year, the corresponding proportion was 37% (SGPALS) Physical inactivity before stroke, stroke severity and fear of falling: ↑↓

Notes: 10MWT = 10 Metre Walking Test; 6MWT = 6 Minutes Walking Test; BESTest = Balance Evaluation System Test; FAC = Functional Ambulation Category; FES-I = Fall Efficacy Scale-International; FSS = Fatigue Severity Scale; HADS = Hospital Anxiety and Depression Scale; IHD = Ischaemic Heart Disease; MoCA = Montreal Cognitive Assessment; MMSE = Mini Mental State Examination; MVPA = Moderate to Vigorous Physical Activity; NIHSS = National Institute of Health Stroke Scale; SwePASS = Swedish Postural Assessment Scale for Stroke; BBS = Berg Balance Scale; SGPALS = Saltin-Grimby Physical Activity Level Scale; TUGT = Timed Up and Go Test
